# Mechanistic insights into the deleterious roles of Nasu-Hakola disease associated TREM2 variants

**DOI:** 10.1038/s41598-020-60561-x

**Published:** 2020-02-27

**Authors:** Raju Dash, Ho Jin Choi, Il Soo Moon

**Affiliations:** Department of Anatomy, Dongguk University College of Medicine, Gyeongju, 38066 Republic of Korea

**Keywords:** Computational biophysics, Protein function predictions

## Abstract

Recently, the critical roles played by genetic variants of TREM2 (Triggering Receptor Expressed on Myeloid cells 2) in Alzheimer’s disease have been aggressively highlighted. However, few studies have focused on the deleterious roles of Nasu-Hakola disease (NHD) associated TREM2 variants. In order to get insights into the contributions made by these variants to neurodegeneration, we investigated the influences of four NHD associated TREM2 mutations (Y38C, W50C, T66M, and V126G) on loss-of-function, and followed this with *in silico* prediction and conventional molecular dynamics simulation. NHD mutations were predicted to be highly deleterious by eight different *in silico* bioinformatics tools and found to induce conformational changes by molecular dynamics simulation. As compared with the wild-type, the four variants produced substantial differences in the collective motions of loop regions, which not only promoted structural remodeling in the CDR2 (complementarity-determining region 2) loop but also in the CDR1 loop, by changing inter- and intra-loop hydrogen bonding networks. In addition, structural studies in a free energy landscape analysis showed that Y38, T66, and V126 are crucial for maintaining the structural features of CDR1 and CDR2 loops, and that mutations in these positions produced steric clashes and loss of ligand binding. These results showed the presence of mutations in the TREM2 ectodomain induced flexibility and caused structural alterations. Dynamical scenarios, as provided by the present study, may be critical to our understanding of the roles of these TREM2 mutations in neurodegenerative diseases.

## Introduction

The accumulation of amyloid-β (Aβ) in brain parenchyma is the main hallmark of Alzheimer’s disease (AD), which results in a slow progressive decline of cognitive function by causing the formation of intracellular neurofibrillary tangles, synapse loss, and cell death^1,2^. Although the precise mechanisms and molecular determinants of this neurodegenerative disease are incomplete, recent whole-genome sequencing studies have demonstrated altered genetic loci including those in TREM2 (Triggering Receptor Expressed on Myeloid cells 2) are associated with a markedly higher risk of progression to AD^3,4^.

TREM2 is a V-type immunoglobulin (Ig) domain-containing transmembrane protein that is expressed in osteoclasts, microglia, alveolar macrophages, and other mononuclear phagocytes^4^. The activation of TREM2 is initiated by anionic lipids, such as bacterial lipopolysaccharide (LPS) and phospholipids, and several putative ligands like apolipoprotein J (ApoJ) and apolipoprotein E (ApoE) have been reported to bind to TREM2^5–7^. Upon ligand binding, TREM2 recruits protein tyrosine kinase SYK through an adapter protein known as DNAX-activating protein of 12 kDa (DAP12), which interacts with the transmembrane region of TREM2^8–10^. This binding is followed by a cascade of signaling events, which include the activations of MAPK and PI3K, and regulates the phagocytosis of cellular debris and the inflammatory responses of microglia^10–12^. During early and mid-term AD, TREM2 plays a protective role^13^, and its overexpression is associated with clearing soluble and insoluble Aβ42 aggregates from brain^14,15^. Furthermore, TREM2 has also been reported to suppress the accumulation and diffusion of Aβ by modulating microglial activation around amyloid plaques^16–19^.

During the past six years, cell biological research studies have suggested links between several TREM2 variants and failure of Aβ clearance, and several rare variants have been shown to be associated with AD progression (Table S1)^20^. Mutations, such as, R47H and R62H variants have been shown to present significant risks of AD, and, as a result, are considered AD-associated variants^21^. Interestingly, at the molecular level, genetic variations in TREM2 have also been linked with frontotemporal dementia (FTD) and Nasu-Hakola disease (NHD), the latter of which is characterized by demyelination, early-onset dementia, and bone cyst lipoma and known to be associated with Y38C, W50C, T66M, and V126G mutations in the ectodomains of TREM2^22–25^.

In this regard, Yeh *et al*. showed that TREM2 variants, including AD-associated (R47H, R62H, and D87N) and NHD linked mutations (Y38C and T66M) reduce binding between TREM2 and its ligands. Moreover, loss of ligand binding was found to be more severe for NHD associated mutations than AD-associated mutations^7^. The present study provides *in silico* insights of the magnitudes of the damaging effects of TREM2 variants, particularly of NHD associated mutations, and provides classical molecular dynamics simulation-based descriptions of the structural dynamic behavior of TREM2 protein in the wild and mutated states.

## Results and Discussions

### Assessment of pathogenicity of TREM2 variants

**Figure 1:**
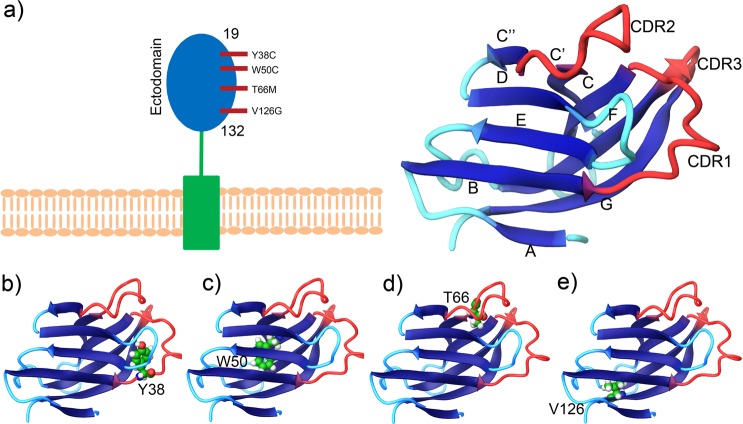
Wild-type and variant structures of the TREM2 ectodomain. Cartoon depiction of the TREM2 wild-type ectodomain showing domain boundaries (**a**). 3-D view of NHD-associated mutated positions (green colored residue), Y38C (**b**), W50C (**c**), T66M (**d**), and V126G (**e**).

Human TREM2 is composed of 230 amino acids and a polypeptide chain that consists of three distinct regions, namely, an N-terminal mature ectodomain (ECD, residues 19–174), a membrane-spanning region (residues 175–195), and a C-terminal cytosolic tail (residues 196–230). The other amino acids, especially residues 1–18 act, as a signaling peptide in the TREM2 signaling cascade. As shown in Fig. 1, the tertiary structure of the TREM2 ECD domain is mainly composed of nine β-strands (βA - βF), which include three major complementarity-determining regions (dubbed CDR loops), that is, CDR1 (residues Pro^37^ to Arg^47^), CDR2 (residues Thr^66^ to Arg^76^), and CDR3 (His^144^ to Glu^117^). Like the other members of the Ig superfamily, ligands bind to TREM2 ECD near apical CDR loops. Previous studies have shown that CDR2 maintains a stable conformation in normal conditions by maintaining H-bonding using the CDR1 loop, which is necessary for ligand interactions^26^. However, genetic variations result in the destabilizations of these loops, and thus, by impairing ligand binding may have deleterious effects. According to X-ray diffraction analysis, the H-bonding network between CDR loops appears to be lost in the R47H variant and result in conformational remodeling of the CDR2 loop. In the present study, we used *in silico* deleterious prediction analysis to re-rank the risk associations of known disease-associated TREM2 variants. Eight *in silico* state-of-the-art-tools were utilized to predict deleteriousness: SIFT (= 0), PolyPhen‐2 (>0.9), PROVEAN (<−2.5), I‐Mutant 3.0 (<−0.5), FATHMM (<−3.0 or >3.0), MutPred (>0.75), CADD (>20), and Condel (>0.8), where parenthesis show the cutoffs used. Of these tools, I-Mutant 3 predicted the highest number of deleterious variants, though all predictions substantially concurred (Fig. S1). In fact, *in silico* predictions of any two tools were found to be significantly associated for most combinations (*P* < 0.0001 by the Student’s *t*‐test). Analysis showed the NHD W50C mutation was the most deleterious by all tools, whereas other NHD variants, including Y38C, T66M, and V126G, were classified as significantly deleterious (*P* = 0.0001) by seven of the eight computational tools. Interestingly, these results are consistent with those of a previous experimental study, in which the Y38C and T66M mutations (located in the CDR1 and CDR2 regions, respectively) were found to be involved in loss of ligand binding. Since NHD variants were found to be more deleterious by *in silico* analysis and experimental findings, we systematically analyzed how these mutations contribute to the pathological behavior of TREM2.

### Changes in the conformational stabilities of TREM2 variants

**Figure 2:**
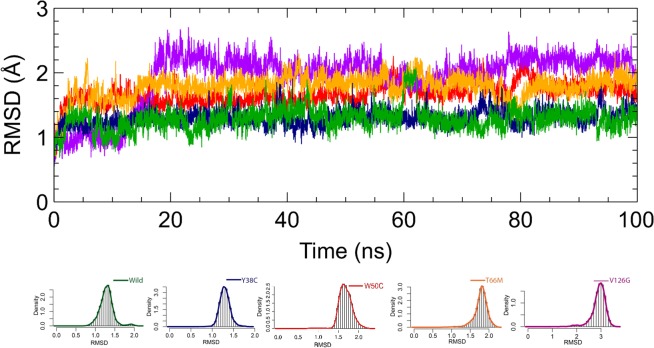
Root-mean-square deviation (RMSD) of C_*α*_ atoms for wild-type and TREM2 variants at 100 ns. Here, the dark green line represents the wild-type, and the blue, red, orange and violet lines represent Y38C, W50C, T66M, and V126G variant RMSDs. In addition, the bottom panel shows density plots for the wild-type and variants and illustrates the distribution**s** of sampled conformations during simulation.

Over past decades, molecular simulation has provided means of characterizing in detail the structural configurations of macromolecules in various environments, as determined by their functions and interactions with other molecular species^27,28^. In this context, molecular dynamics simulations of wild-type and Y38C, W50C, T66M, and V126G variants were conducted for 100 ns to access their structural dynamic and stability characteristics. The conformational stabilities of wild and variant types during simulations were analyzed by calculating RMSD values for the backbones of all proteins from starting structures (Fig. 2**)**. RMSD analysis revealed that the wild-type and Y38C variant achieved equilibrium after 5 ns and that equilibrium states were maintained until the end of simulations, whereas the three other variants achieved equilibrium after 20 ns. Furthermore, wild-type and Y38C structures showed similar deviations from their starting structures at 100 ns, which resulted in backbone RMSD values of ~1.4 to 1.7 Å during simulations. However, W50C, T66M, and V126G structures showed significantly different deviations from wild-type and Y38C structures (range ~1.9 to 3 Å). After the relaxation period, the magnitudes of fluctuations of the Y38C, W50C, and V126G variants were stably maintained^29^. However, the RMSD of the T66M variant reduced at 80 ns but then remained stable from 84 ns to the end of the simulation, indicating a major conformational change occurred. A similar pattern was also observed for the W50C variant. Overall, RMSD analysis suggested that the trajectories produced provided an appropriate basis for further analysis.

**Figure 3:**
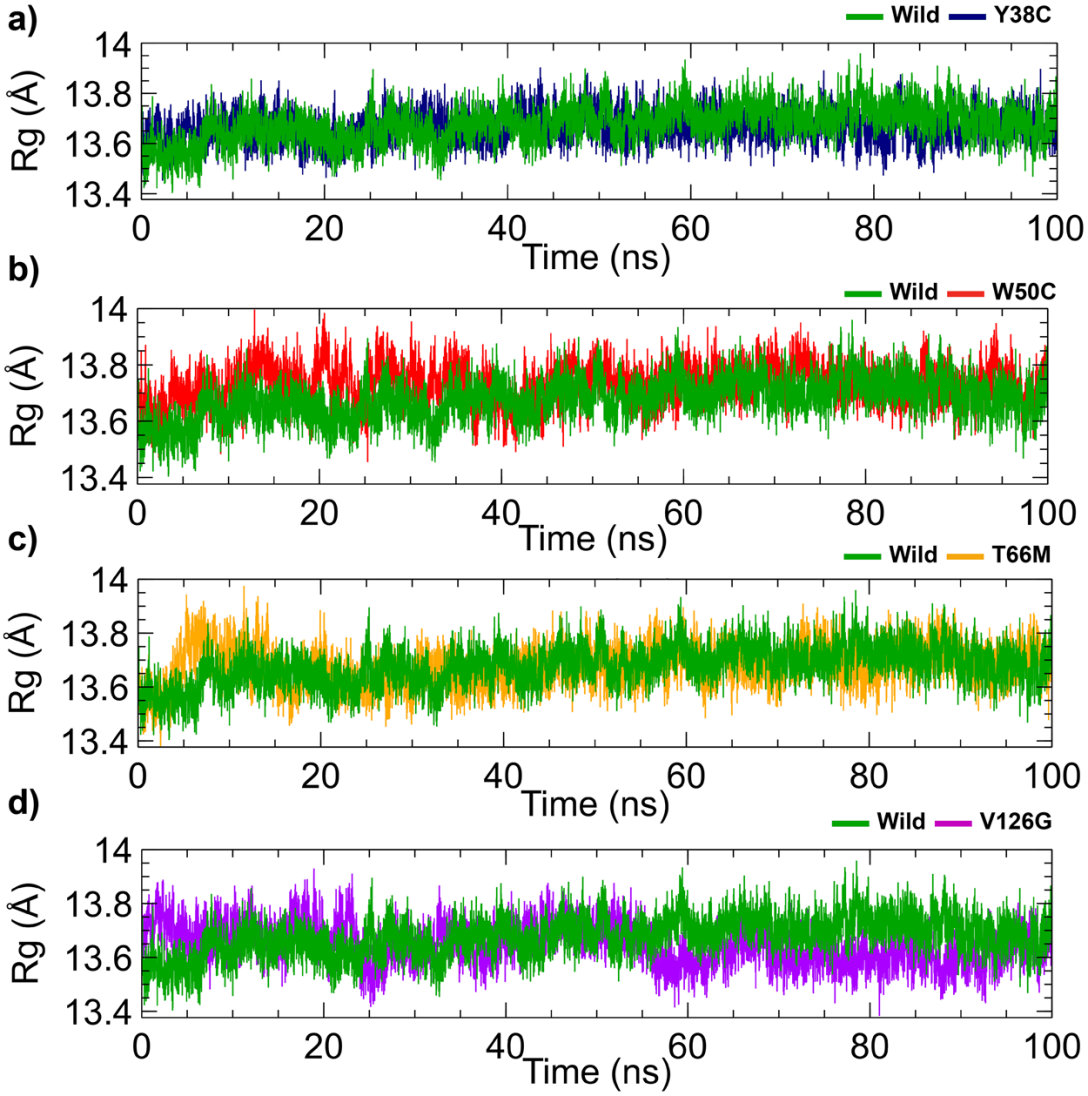
Radius of gyration of the Y38C (**a**), W50C (**b**), T66M (**c**), and V126G (**d**) variants versus wild-type TREM2. Here, the dark green line represents the wild-type and blue, red, orange, and violet lines represent the Y38C, W50C, T66M, and V126G variants, respectively.

**Figure 4:**
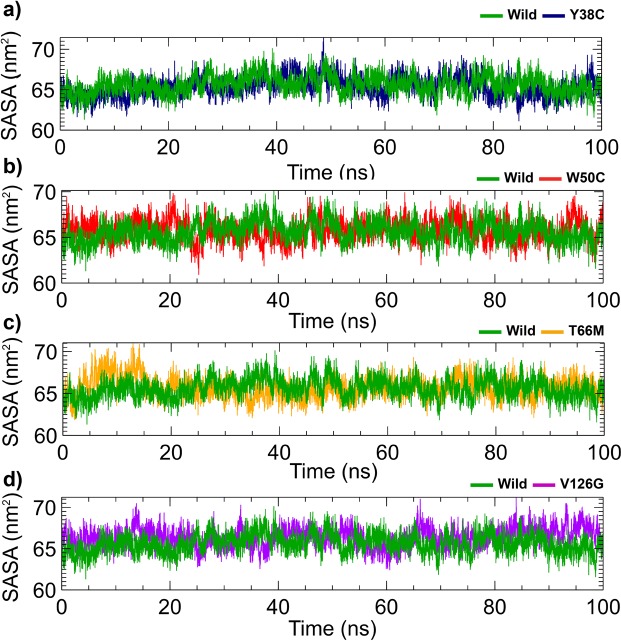
Changes in total solvent accessible surface areas (SASA) of the Y38C (**a**), W50C (**b**), T66M (**c**), and V126G (**d**) variants as compared with wild-type TREM2. Here, the dark green line represents the wild-type and blue, red, orange, and violet lines represent Y38C, W50C, T66M, and V126G variants, respectively.

Conformational in changes in variants as compared with the wild-type were evaluated using radius of gyration (Rg) **(**Fig. 3**)**, which describes overall dimensions of protein structures in terms of compactness (higher Rg values represent lower compactness)^30,31^. As shown in Fig. 3a and c, the overall dimensions of Y38C and T66M were similar to those of the wild-type, whereas V126G (Fig. 3d) showed increased compactness from 50 ns to the end of the simulation. On the other hand, W50C (Fig. 3c) had a higher Rg than the wild-type. SASA provides a measure of solvent accessibility, and SASA profiles were similar to Rg profiles for variants and the wild-type. The wild-type and Y38C, W50C, and T66M variants showed similar deviations from their initial structures (Fig. 4a–c), but V126G showed an increase in SASA after 50 ns (Fig. 4d), which indicated bloating of its structure as compared with the wild-type^32^. Although RMSD analysis showed conformational alterations in T66M and V126G variants versus the wild-type, only the V126G variant showed conformational alterations by Rg and SASA. T66M showed a decrease in RMSD before 80 ns, and Rg and SASA values also reduced at this time point. Summarizing, Rg, SASA, and RMSD analyses **(**Table S1**)** showed that the Y38C, W50C, T66M, and V126G variants modulated protein dimensions, which in turn suggested misfolding and changes in protein-protein interactions^33^.

**Figure 5:**
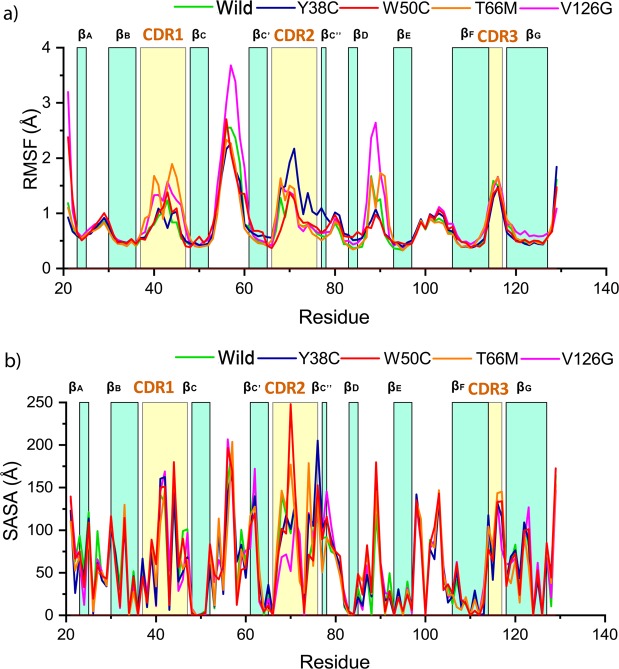
Investigation of the local structural effects of mutations. Root mean square fluctuations of C_α_ atoms for wild-type TREM2 and the four variants (**a**). SASA values were computed on a per residue basis for the wild-type and TREM2 variants (**b**). In all plots, residues index is level with colored bar, representing the structural propensity. In each graph, the dark green line represents the wild-type, while blue, red, orange, and violet lines represent the Y38C, W50C, T66M, and V126G variants, respectively.

Figure 5 shows RMSF values for protein backbones and depicts local changes in protein structure caused by mutations. Local residue fluctuations in proteins underlie biological functions, as the many functional sites in proteins are uniquely coupled with structural fluctuations^34–36^. As represented in Fig. 5a, TREM2 contains ten loops, including three CDR loops, which all fluctuated throughout simulations. Degrees of fluctuation were greater in variant structures than in the wild type. Interestingly, Y38C and T66M variants showed high levels of fluctuation in CDR1 and CDR2 regions, respectively. The V126G variant showed increased local flexibility, especially in the regions including residues 50 to 60 and 85 to 95 and in the CDR1 loop and N-terminal tail. On the other hand, no significant changes were observed for the W50C variant as compared with the wild-type with the exception of residues 86 to 93, which had a lower RMSF value, suggesting conformational changes in this region. For more insight, SASA values were calculated per residue for the wild-type and variants. Variant structures showed an increase in residual SASA values, especially W50C and T66M in CDR1 and CDR2 regions (Fig. 5b). SASA deviations were also observed for Y38C and V126G variants in the CDR1 and CDR2 loop regions. SASA per residue values reflect structural conformational changes at protein surfaces; lower values mean residues are buried inside globular folded conformations^37–39^. This analysis together and the RMSF study suggest that the Y38C, W50C, T66M, and V126G increase solvent exposure, modulate protein-protein interactions, and possibly increase aggregation propensity^40–42^.

### Effects of mutations on protein dynamics

**Figure 6:**
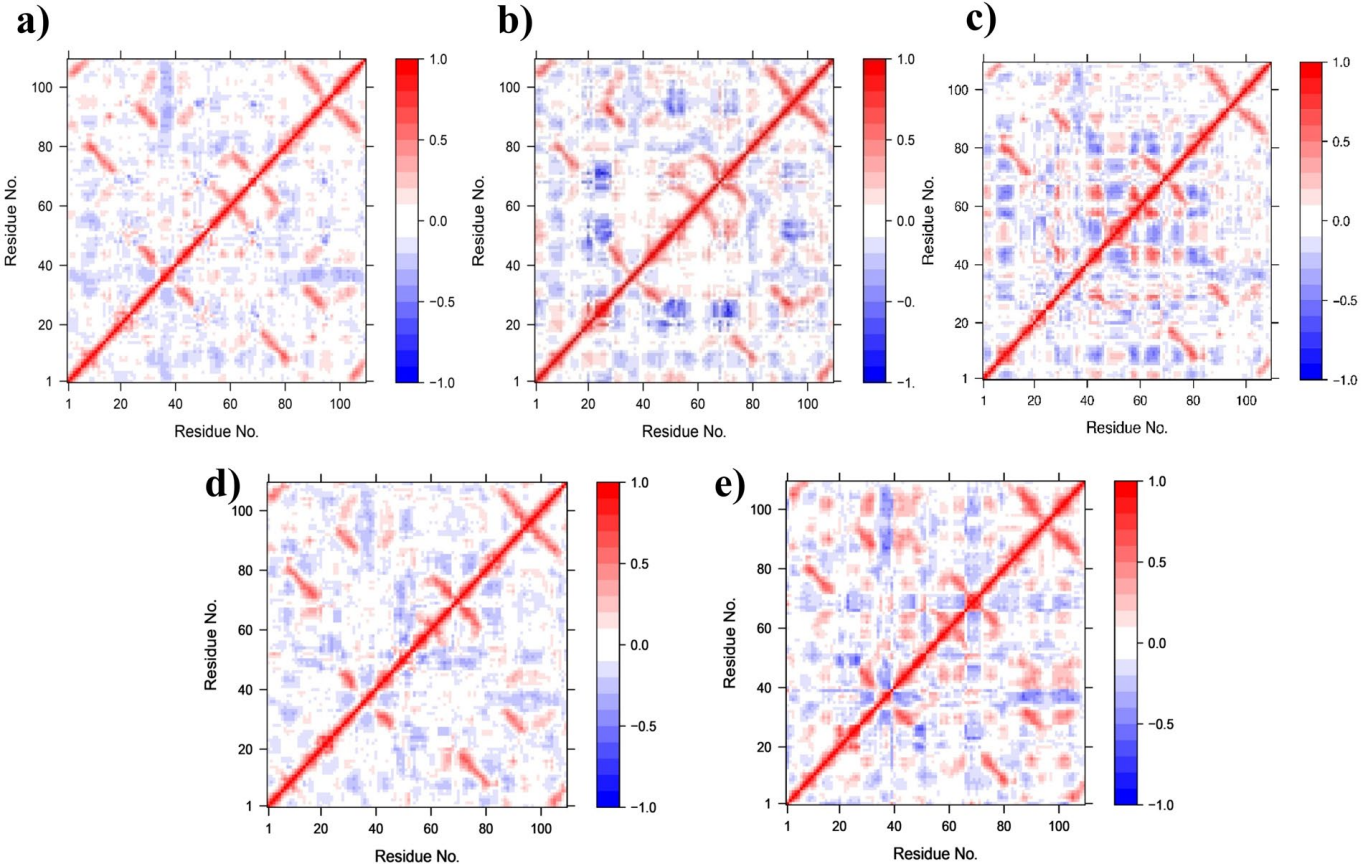
Calculated dynamic cross-correlation matrix of C_α_ atoms around their mean positions for 100 ns molecular dynamics simulations. Extents of correlated motions and anti-correlated motions are color-coded from red to blue, which represent positive and negative correlations, respectively. Wild-type (**a**), Y38C (**b**), W50C (**c**), T66M (**d**), and V126G (**e**).

In different functional states, proteins undergo conformational transitions due to global domain motions facilitated by the collective motions of backbone atoms^43^. Generally, the residues located in the regions with secondary structures move in a concerted manner^44^. For additional insight into the dynamics of wild-type TREM2 and its variants, dynamic cross-correlation maps (DCCM) and principal component analyses were performed on the MD trajectories of different systems. DCCM addresses the concerted motions of protein residues and highlights strongly correlated motions between specific residues in red (Fig. 6), and conversely, highlights highly anti-correlated motions in blue. Analysis revealed that as compared with the wild-type, the Y38C, W50C, T66M, and V126G mutations induced completely different motions. Y38C (Fig. 6b) produced significant anti-correlative motion between residues 17–27 (CDR1 loop, residues 37 to 47) and residues 45 to 56 (CDR2 loop, residues 65 to 76), and 65 to 74 (residues, 85 to 94). The T66M variant (Fig. 6d) slightly reduced the degrees of correlated and anti-correlated motions observed in the wild-type, although no significant correlation has been seen in the DCCM analysis. The W50C variant exhibited correlative motions not observed in Y38C, T66M, or V126G variants, and showed strong anti-correlative motions between residues 5 to 11 (residues, 25 to 31) and residues 38 to 46 (residues, 58 to 66), 56 to 60 (residues, 76 to 80), 62 to 67 (residues, 82 to 87), 73 to 76 (residues, 93 to 96), respectively (Fig. 6c). Furthermore, significant positive correlations were observed between region 40 to 46 (residues, 60 to 66) and 63 to 69 (residues, 83 to 89), the latter of which showed anti-correlative motions with residues in CDR2 loops 50–56 (residues, 70 to 76) and the region including residues from 77 to 85 (residues, 97 to 105). For the V126G variant (Fig. 6e), results showed mutation increased correlative intra-residue movement, specifically between the loop residues, 34 to 40 (residues, 54 to 60) and the c-terminal portion 80 to 105 (residues, 100 to 125). A negative correlation was also observed in the CDR1 and CDR2 loop regions, suggesting the mutation had a deleterious effect on TREM2 functions.

To further support the changes on the protein dynamics, principal component analysis (PCA) was conducted based on C_α_ atoms (Fig. S2). PCA analysis, which reduces the complexity of collective motions is usually performed to obtain insight of the dynamics and mechanical properties of simulated systems^28^. Furthermore, the dynamics of two different proteins are best compared by characterizing their phase space behaviors, which are directly associated with protein stability and function^45,46^. We described the overall combined motions of C_α_ atoms of protein structures using eigenvectors of covariance matrix, which is correlated with the coincident eigenvalues. Trace values for the wild-type and the Y38C, W50C, T66M, and V126G variants were found to be 46.707 Å^2^, 47.487 Å^2^, 58.94 Å^2^, 49.822 Å^2^, and 99.309 Å^2^, respectively, which suggested that mutations induced collective flexibility. V126G had a higher trace value than the wild-type or Y38C, W50C, or T66M variants, although its Rg value reduced after 50 ns of simulation. This observation suggests V126G exhibits significantly greater protein local flexibility rather than overall flexibility, as can be seen in the RMSF plot **(**Fig. 5**)**, which shows greater flexibility in residues 50 to 60 and 85 to 95. To investigate further, three PCAs were considered for further analysis. Resultantly, simulated systems of the wild-type and Y38C, W50C, T66M, and V126G variants showed variances of 19.81%, 33.43%, 24.50%, 20.94%, and 29.67%, respectively, for the first three PCAs. The motions of different proteins can be visualized using projections of trajectories in phase spaces of the first two principal components (PC1, PC2), whereas variants show different patterns of conformational spaces, indicating significant changes in protein conformations. Using these projections, Y38C showed a uniform conformational distribution in a larger phase space, indicating greater flexibility, than the wild-type (Fig. S2b). The T66M variant also showed conformational transitions during simulation that indicated less flexibility over time (Fig. S2d). On the other hand, the W50C and V126G variants exhibited higher periodic jumps, indicating greater fluctuations than the wild-type and the occupation of more phase space (Fig. S2c and e).

**Figure 7:**
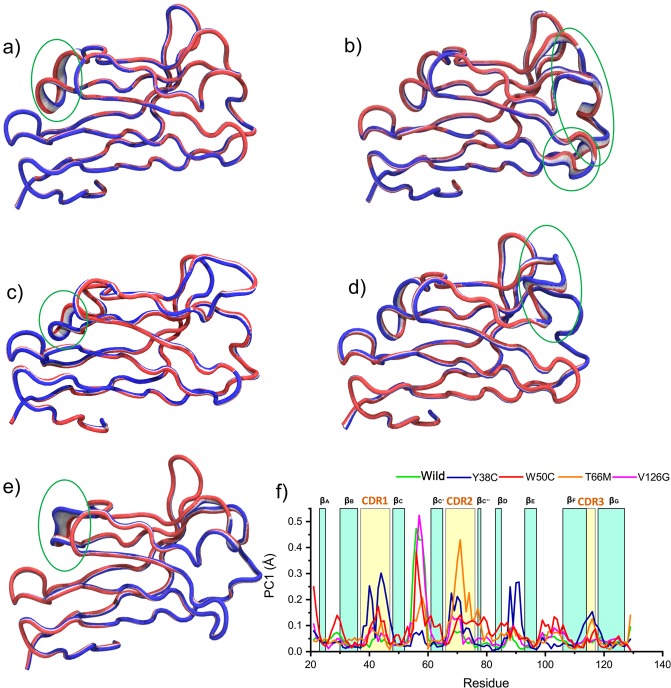
Representation of atomic displacements of PCA1 as determined by simulations of wild-type TREM2 (**a**), and of the Y38C (**b**), W50C (**c**), T66M (**d**), and V126G (**e**) variants, showing significant movement differences. The green circles indicate regions of greater flexibility. Residue-wise loadings for PC1, where the dark green line represents the wild-type and blue, red, orange and violet lines represent the Y38C, W50C, T66M and V126G variants (**f**).

The variations in atomic movement were presented by all first PCAs (Fig. 7**)**, and PC1 exhibited the most dynamic motion. In the figure, the displacement of atoms is denoted by wide tubes, whereas narrow tubes mark regions that remained rigid during the simulation. Structural deviations in the wild-type and in variants were obvious for first PCAs (Fig. 7**)**, in which the contributions of residues to first principal components are represented in the plot rendered in the bottom panel (Fig. 7f), which describes residual mobilities during the simulation. As shown by the figure, Y38C induced flexibility in several loops of TREM2, including CDR1, CDR2, and CDR3 (Fig. 7b), whereas T66M induced significant flexibility in CDR2 (Fig. 7d**)**.

Conformational changes in the wild-type structure were observed in the loop region, including residues 47 to 60, and this region also exhibited significant changes in the W50C variant (Fig. 7c and e). The W50C variant also exhibited more residual mobility in the CDR1 loop than the wild-type. Interestingly, V126G induced the highest residual mobility in the region from 47 to 60, which is consistent with RMSF and DCCM results and supports mutation-induced local conformational changes.

### Mutation-induced conformational changes

Although MD simulations well explained conformational changes in protein structures, we also investigated mutation-driven changes in secondary structures. RMSF, DCCM and PCA analysis showed Y38C and T66M variants exhibited considerable flexibility in CDR regions that might alter structural orientations in these regions. Therefore, variations in secondary structural elements for wild-type and variant proteins during simulation were characterized using Schrödinger 2017–1 simulation interaction diagrams (LLC, New York, NY, USA)^47^. During simulation, the appearance of secondary structural elements determines protein structural flexibility, for example, conformations such as α-helices and β-sheets are naturally more rigid than coil or turn conformations^28^. Total percentages of secondary structural elements in total simulation time are shown in Figs. S3,S4 to S8. Interestingly, variants increased total secondary structures as compared with the wild-type. Moreover, the CDR1 (residue index 17–27) and CDR2 (residue index 46–57) regions gained a short α-helix conformation in variant structures, though the percentage of helix formation was greater in Y38C (Table S2). During simulation, Y38C showed higher α-helix propensity in the CDR1 loop from 20 to 40 ns, although α-helix formation was observed to persist over the entire simulation (Fig. S5). On the other hand, W50C and V126G variants increased the total secondary structure elements in the TREM2, and increases in α-helix conformation in the CDR2 region was greater in W50C and V126G than in Y38C and T66M. The W50C variant only showed α-helix formation in CDR1 loop, but its percentage was less than that of Y38C and T66M. Furthermore, the W50C variant demonstrated significant conformational changes in the region residue 48 to 64 (mainly in β-strands, βc and βc′) within the simulation time 68 to 78 ns (Fig. S6). Notably, the residues of βc and βc′ strands are located near the W50C mutation site, where formation and reduction of β-strand propensity were observed, as a result, the RMSD pattern was seen to change in this time point. In addition, T66M had low RMSD values at simulation times <80 ns. Interestingly, during this period, T66M showed aggressive α-helix formation in the CDR2 loop, which supported the dramatic behavior of T66M during the simulation (Fig. S7). Previous studies on AD associated variants have shown that substitution of R47 by H47 facilitates CDR2 loop remodeling to a short α-helix, which contributes to loss of ligand binding^26^. In summary, our results suggest that the variants examined facilitate conformation remodeling of CDR2 loop and of CDR1 regions.

### Mechanistic insights into conformational remodeling

Intra-residue H-bonding influences the secondary structures of biological macromolecules^48^. Thus, H-bond occupancy analyses within CDR1 and CDR2 loops of the wild-type and variants types were undertaken as both loops undergo conformational remodeling (Fig. S9). As shown in Fig. S9, mutations induced diverse intra and inter loop H-bond interactions. Furthermore, these loops were identified as flexible regions by RMSF and PCA studies, indicating that increasing flexibility by mutations render residues liable to different interaction networks. According to the RMSF study, the substitution of tyrosine with cystine at the 38^th^ position induced high flexibility next to D39 and facilitated H-bond interactions within the CDR1 loop, especially with M41 and H43 (Fig. S9a). H-bond occupancy between D39 and H43 in Y38C variant was maintained at 12.34% during simulation, whereas it was 3.91% in the wild-type. Furthermore, D39 maintained 6.6% H-bond occupancy with M41, but this interaction was absent in the wild-type. This observation indicates that maintaining intra-loop H-bonds between D39 and M41 or H43 may facilitate short helix formation of the CDR1 loop in Y38C. In the case of the CDR2 loop, the Y38C variant showed less H-bonding between S73 and W70, L72, and S75 than in the wild-type. In addition, Y38C exhibited two new H-bonds between T66 and S73 and between S73 and L69, which were lacking in the wild-type structure (Fig. S9b).

Previous studies have shown that conformational changes of the CDR2 loop are facilitated by π-π stacking interactions between the imidazole moieties of H47 and H67, which cause H67 to swing by ~180 °^26^. When we investigated the inter-loop interaction between CDR1 and CDR2 we found that Y38C increased H-bonding between H47 and H67 (Fig. S9c), which indicated conformational changes in the CDR1 loop.

Similarly, the T66M variant showed significant local fluctuations in S65 and H67 residues as compared with the wild-type; H67 maintained H-bonding with L72 by >20%. Interestingly, mutation at T66 increased H-bonding capacity with R47 versus the wild-type. However, unusual H-bonding between R47 and S65 was observed after replacing M66 in T66M, which possibly contributed to conformational changes of CDR1. Nevertheless, the interaction between M66 and R47 dominated, and as a result, the degree of helix formation in the CDR1 loop was observed to decrease subsequently during the simulation (Fig. S9c).

The W50C mutation reduced intra-loop H-bonding in the CDR1 loop versus the wild-type, and K42 maintained H-bonding only with the D39 residue with an occupancy of 39.41%. Furthermore, the W50C mutation changed the H-bonding pattern in the CDR loop, where it increased the interaction between T66 and S73 and between H67 and L72. Additionally, the interaction between T66 and R47 was also greater in W50C, in which R47 also interacted with the H67 residue with an occupancy of 8.22%. On the other hand, V126G mutation reduced H-bonding between R47 and T66 and induced interaction between R47 and N68, which might facilitate conformational CDR1 loop changes. Besides, conformational changes in CDR2 loop could be induced by numerous H-bonding interactions between H67 and M41, H43, and T66.

**Figure 8:**
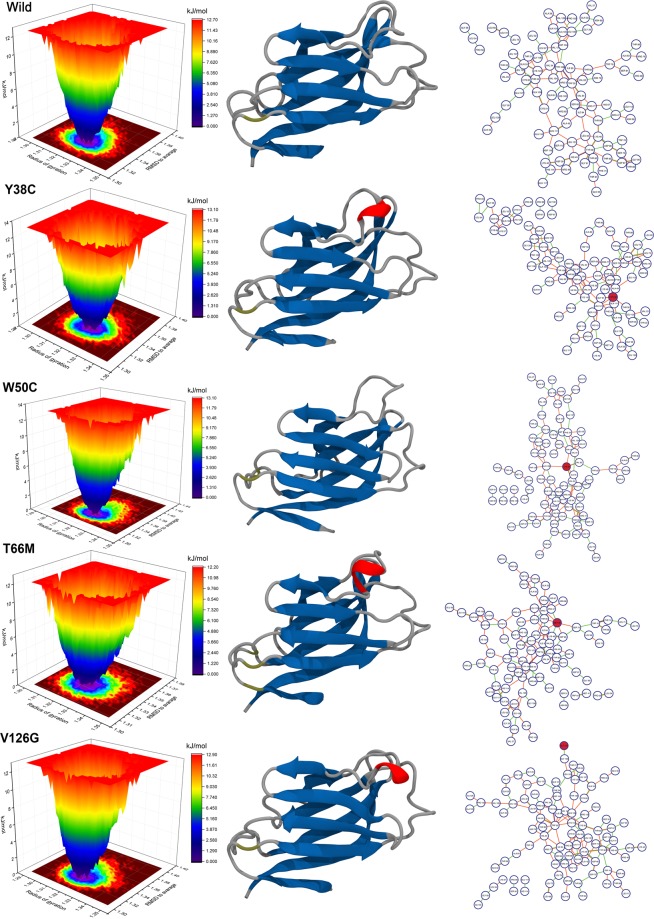
Free energy contour maps derived from radius of gyration and RMSD values, where the dark blue color area indicates lower energy. Each graph accompanied by a corresponding 3-D structure of lowest energy and a residue interaction network. Nodes in networks represent residues, whereas edges represent interactions. Edge colors signifying indicate interaction types, i.e., green = hydrogen bond, orange = van der Walls contact, cyan = salt bridge, blue = pi-pi stacking, and pink = disulfide bridge.

In order to validate our H-bonding findings, we performed additional free energy landscape (FEL) analysis to select the most stable conformer from the simulation ensemble. FEL results are rendered in Fig. 8, where different colors represent structures with different energies. Lower energy conformations are colored blue and are usually more stable than other conformations (red) generated by simulation^49^. The thermodynamic stabilities of proteins are represented by depths of energy minima, and protein kinetic stabilities are indicated by the height of barriers between energy minima.

The depths of energy minima may also suggest conformational changes, where the width of an energy minimum associated with the number of conformational ensemble within the energy well^50^. As shown in Fig. 8, the region of conformational space corresponding to the basin changed in the wild-type and in variants, which indicated that the overall conformational stability TREM2 is affected by mutations. In order to understand the disordered state, the conformation from the favorable energy minima has been visualized. We also analyzed the intra-residue H-bonding network**s** using residue interaction network analysis. The lowest minima in the wild-type trajectory was found to have average Rg and RMSD values of 1.33 and 1.32 nm, respectively, which exhibit no conformational changes in the CDR1 or CDR2 loops.

In detailed structural view, T66 was found to interact with R47 by forming two H-bonds. A short helix was found in the CDR2 loop of the most stable conformer of variant trajectories. In Y38C, M41 maintained a H-bond with D39, and R47 H-bonded with H67 and L72, while in T66M, R47 maintained a π-π stacking interaction with H67. Intra and inter-loop H-bonding analysis showed a similar residual interaction in W50C, but when whole trajectory systems were considered, the interaction between D39 and H43 was maintained in the residual interaction network (Fig. S10c). On the other hand, the V126G variant showed unusual H-bonding between residues 66 and 67 and between R47 and S65. Although no conformational change in the CDR1 loop was visualized, interaction between D39 and M43 was also seen within the CDR1 loop. These observations were consistent with the residual interaction network**s** generated from total MD trajectories (Fig. S10).

Computational analyses in mutational landscapes, particularly MD simulations, have proven to be crucial tools in terms of deciphering the structural basis of protein aggregation and misfolding at the molecular level^51^. MD simulation provides a complete understanding of the phenotypic expressions of mutations by generating detailed information on conformational and structural consequences at a reasonable quality and adequate time scale^52^. Several recent studies have investigated the correlation between experimental studies and MDS analysis, and have suggested MDS studies are helpful for uncovering underlying mutation-associated disease mechanisms, especially in the context of neurodegeneration^40,53–57^. It has been suggested in previous MD simulation studies that R47H is responsible for loop distortions in TREM2^58^ and to induce flexibility, particularly in CDR2 loops^59^. Furthermore, an X-ray crystallography study on the R47H mutation concluded that it mediated CDR2 remodeling by causing short helix formation in the loop region that ultimately abolished the ligand interaction and caused loss of ligand binding^26^.

Consistent with the mechanisms deemed responsible for loss of function by AD-associated variants, as described in studies mentioned above^26^, NHD mutations in the present study also showed significant structural alterations during molecular dynamics simulations, especially in the loop regions. It has been demonstrated mutations involving the substitution of an amino acid with different properties can disrupt molecular function by disturbing domain organization^28,60^. Likewise, in the present study, substituents at the 38 and 66 positions of TREM2 were found to induce effects that differed from those of native residues, and consequently to cause steric clashes between junctions of the CDR1 and CDR2 loops. Other W50C and V126G mutations had similar effects, and V126G was found to induce steric clash in CDR1. As was revealed by H-bond analysis, all of these mutations altered H-bonding patterns in CDR loops in the most native conformational states and in overall MD ensembles. Sudom *et al*. reported residues S65, T66, H67, N68, and H67 were critical for maintaining conformation stability of the CDR2 loop by forming a H-bonded network with residue R47 in the CDR1 loop^26^. Similarly, NHD mutations caused also deleterious effects similar to those caused by the AD-associated R47H mutation, which indicates this H-bonding network is important for maintaining the stabilities of CDR1 and CDR2 loops. Kober *et al*.^61^ suggested that NHD mutations might cause conformational changes of buried TREM2 residues, promote misfolding, and inconsistently impact TREM2 surface expression and aggregation. In particular, our simulation-based study showed that the Y38C, W50C, T66M, and V126G mutations cause CDR aggregations by changing secondary structural preferences, and hence, contribute to NHD-associated loss of function.

## Conclusion

The effects of genetic variants on TREM2 loss of function is crucial to understanding its involvement in late-onset Alzheimer’s disease (AD). Using molecular dynamics simulation, this study presents novel findings on the deleterious roles of NHD variants, which were found to promote structural alterations in the ectodomain of TREM2. The Y38C, W50C, T66M, and V126G mutations examined all increased flexibility and altered hydrogen bonding patterns, and Y38C, T66M, and V126G induced structural remodeling in the CDR1 and CDR2 loops by inducing steric clashes. This study supports previous findings and provides additional insight of the mechanism responsible for loss of ligand binding, which is critical to our understanding of the role of TREM2 in neurodegenerative diseases.

## Methods

### Prediction of pathogenicity from *in silico* tools

The deleterious effects of selected variants were characterized using several *in silico* bioinformatics tools and information retrieved from the NCBI dbSNP^62^. Eight tools were utilized, namely, Sorting Intolerant From Tolerant (SIFT)^63^, Polymorphism Phenotyping v2 (PolyPhen-2)^64^, Functional Analysis through Hidden Markov Models (FATHMM)^65^, PROVEAN^66^, Mutpred^67^, I‐Mutant 3.0^68^, Combined Annotation Dependent Depletion (CADD)^69^, and Condel 2.0^70^. SIFT using sequence homology approaches to predict deleterious effects, whereas PolyPhen-2 uses the position‐specific independent counts (PSIC) scoring method and the Bayesian classifier. Pathogenicity prediction by FATHMM is typically accomplished using hidden Markov models (HMMs), and I-Mutant 3.0 uses a support vector machine algorithm to calculate the stabilities of variant protein structure based on free energy changes. Mutpred predicts gain and loss of 14 different structural and functional properties of proteins caused by mutations. CADD considers protein level scores derived by SIFT and Polyphen analysis and a broad range of data, including functional genomics data, to predict the pathogenicities of nonsynonymous mutations. On the other hand, Condel uses a consensus approach to predict SNP impact by integrating Log R Pfam *E-value* (LogRE)^71^, MAPP^[8,19,72^, Mutation Assessor^18,73^, Polyphen2 (PPH2)^64^, and SIFT^63^.

### Preparation of the simulation system

The three-dimensional crystal structure of the ectodomain of TREM2 (PDB id of 5ELI^61^) was retrieved from the protein databank (http://www.rcsb.org/pdb)^74^. The structure of wild-type TREM2 was initially prepared by adding bond orders, hydrogens, and charges and refined by removing water molecules and optimizing it at neutral pH. The structure was further fixed by correcting for asparagine amide groups, some thiol and hydroxyl groups, protonation states of glutamic acids, aspartic acids, and histidines. In order to adjust heavy atom Root Mean Square Deviation (RMSD), minimization was applied by using Optimized Potentials for Liquid Simulation (OLPS3) force field down to 0.30 Å. Y38C, W50C, T66M, and V126G variant structures were constructed by computational mutagenesis using the Mutate Residues script from Schrödinger suite 2017-1 (LLC, New York, NY, USA)^75^.

Additional short molecular dynamics refinement simulation was performed to better resolve the structure**s (**wild-type TREM2 and those of the Y38C, W50C, T66M, and V126G variants). Thus, molecular dynamics simulation was conducted by applying YAMBER3 force field^76^ for 500 ps at pH 7.4 and 298 K at a solvent density of 0.997 g/cc. The simulation was run using YASARA software by a default md_refine macro, and the final structure**s** were selected based on the lowest free energy energies.

### Molecular dynamics simulation

Molecular dynamics simulation was used to study the changes in the dynamic behaviors of protein caused by mutations using the Desmond module of Schrödinger suite 2017-1 (LLC, New York, NY, USA)^77,78^. Here, OLPS3 all-atom force field was utilized to visualize molecular behavior^79–81^. Structures were prepared for wild-type TREM2 and the Y38C, W50C, T66M, and V126G variants solvated in the presence of explicit solvent in a triclinic periodic boundary box. Each system was submerged to a Monte-Carlo equilibrated TIP3P solvation model extending to ~10 Å in each direction, which is known to provide best experimental outputs^82^. Additional counter ions were added to the water model to neutralization the system. Physiological condition and ionic strength of the solvent system were maintained by maintaining a default salt (NaCl) concentration of 0.15 M in the simulation box. The default relaxation protocol^83,84^ was used to relax the 8-stage system. Followed by Brownian dynamics, the second stage simulation was started at 10 K in an NVT ensemble for 12 ps with restraints on solute heavy atoms. Third stage simulation was done for 12 ps in the same ensemble at 10 K with restraints on solute heavy atoms. The fourth stage was begun by running 12 ps simulation under the NPT ensemble at 1 bar pressure while maintaining solute heavy atom restraints. In the fifth stage, protein cavity was solvated using the solvate pocket script. Stage 6 involved 12 ps simulation in an NPT ensemble with restraints on solute heavy atoms, and stage 7 involved a 24 ps simulation with no restraints on solute heavy atoms (both stages were performed at 300 K at 1 bar. Finally, a 100 ns MD simulation was performed for each system with no constraints applied, and resulting trajectories were subjected to further analysis. Throughout the simulation, RESPA^85^ integrator (a motion integration package) was employed with 2 fs as inner time step with the M-SHAKE^86^ algorithm to constrain all covalent bonds connecting hydrogen atoms. The Particle Mesh Ewald method was used to calculate long-range electrostatic interactions and 9.0 Å was used for short-range electrostatic contacts; uniform density approximation was select for the cutoff of long-range van der Waals (VDW) interactions. Conditions during simulations were maintained by Nose–Hoover thermostats^87^ at 300 K and 1 atmosphere using the Martyna–Tobias–Klein method^88^.

Resultant trajectories were used to evaluate protein conformational changes and stabilities using Root Mean Square Deviation (RMSD), Root Mean Square Fluctuation (RMSF), and SSE (Secondary Structure Elements) in the Simulation Interactions Diagram panel of Schrödinger 2017-1 (LLC, New York, NY, USA). Radius of gyration (Rg), Solvent Accessible Surface Areas (SASA), and H-bond occupancies were calculated using VMD software^89^. Dynamic cross-correlation maps were generated from trajectories to explain time-correlated protein motions, which were assembled using Bio3D^90^ software by R programming. Time-correlated information between protein atoms i and j (c_ij_) was represented as a matrix in DCCM, which was obtained using the following expression:

$$DCC{M}_{i,j}=\frac{{\mathop{\to }\limits_{d}}_{i}.\,{\mathop{\to }\limits_{d}}_{j}}{\sqrt{{d}_{i}^{2}|{d}_{j}^{2}}}$$

Displacements between current and average positions of atom i and j are represented by *d*, and mean times overall trajectories are represented by the angled brackets. Calculated values in DCCM ranged between −1 and +1, which denoted negative and positive correlations, respectively. Principle Component Analysis (PCA) was further utilized to describe the collective motions of TREM2 variants^44^. PCA eigenvectors were calculated by superimposing atomic coordinates on reference structures without translational or rotational movements. Eigenvectors represented mean square displacements (MSD) of atoms and were associated with eigenvalues. Mathematical details have been previously described in detail^91,92^.

### Free energy landscape (FEL)

The free energy landscape technique is used to map all possible conformational changes of macromolecules using energy levels derived from the spatial dispositions of interacting molecules^93,94^. For FEL analysis, Gibb’s free energy is calculated as a function of protein enthalpy and entropy and also identifies conformational states related to protein structure-function correlations. In the present study, the energy bases of conformational diversity in various TREM2 structures were investigated using the following equation:

$${{\rm{G}}}_{{\rm{i}}}=-{k}_{B}Tln(\frac{{N}_{i}}{{N}_{max}})$$

where k_B_ is Boltzmann’s constant, *T* is the temperature (which was set at 300 K), *N*_*i*_ is the population in bin *i* and *N*_max_ is the population in the most populated bin. An artificial barrier scale is set to the bin with no population with the lowest provability. Color-code modes were used to display different energy levels.

### Residue interaction network

To analyze the Residue Interaction Network (RIN), the most stable 3-D coordinates of the wild-type and of variants were transferred to the RING server^95^, which provides intra-residue interactions in the exhaustive network view. In the network model, protein residues are represented by nodes and interaction modes as edges. Results from the RING server were then processed using Cytoscape to construct interactive RIN 3.2.1^96^ using the plug-in RINalyzer. Types of interactions are described by dashed or dotted edges, and salt bridge, H-bonding, and van der Waal interactions. In addition, residual interaction networks were constructed by considering the whole trajectories of 100 ns molecular dynamics simulations. For that 1000 representative structures with an interval of 100 ps from each simulation system were visualized and analyzed using a combination of UCSF Chimera^97^, structureViz^98^, and Cytoscape^99^.

## Supplementary information

Supplementary data.
