# Supplementary material for: Mechanistic insights into the deleterious roles of Nasu-Hakola disease associated TREM2 variants
Source: Sci Rep. 2020 Feb 27;10:3663. doi: 10.1038/s41598-020-60561-x (PMC7046722; doi:10.1038/s41598-020-60561-x)
Supplement: Supplementary file 1 — Supplementary data. [file 41598_2020_60561_MOESM1_ESM.docx]

**Supplementary data**

**Mechanistic insights into the deleterious roles of Nasu-Hakola disease associated TREM2 variants**

Raju Dash^1^, Ho Jin Choi^1^, Il Soo Moon^1^*

^1^Department of Anatomy, Dongguk University Collage of Medicine, Gyeongju 38066, Republic of Korea

* Correspondence:

Il Soo Moon

Email: [moonis@dongguk.ac.kr](mailto:moonis@dongguk.ac.kr)

**Table S1:** List of TREM2 variants associated with neurological disorders with their details regarding experimental evidence of loss of function as well as *in silico* deleterious prediction.

| Substitution | Experimental Evidence | MDS | Disease  Association | References | *In silico* SNP analysis | | | | | | | |
| --- | --- | --- | --- | --- | --- | --- | --- | --- | --- | --- | --- | --- |
|  |  |  |  |  | SIFT | PolyPhen-2 | PROVEAN | I-mutant 3 | FATHMM | MutPred | CADD | Condel |
| A28V | N/A | N/A | FTD | ^1^ | 0.14 | 0.08 | -0.87 | 0.65 | -0.08 | N/A | 3.39 | 0.25 |
| Y38C | LB | N/A | NHD ^2^ | ^3^ , ^4^ | **0** | **1** | **-8.80** | **-1.23** | -0.34 | **0.72** | **26.3** | **0.94** |
| D39E | N/A | N/A | AD ^5^ |  | 0.01 | 0.49 | -1.25 | 0.1 | 2 | N/A | **24.2** | 0.57 |
| R47H | LB | N/A | AD ^2^ | ^3^ | 0.02 | **0.98** | **-2.56** | **-1.87** | -0.21 | 0.31 | **24** | **0.82** |
| W50C | N/A | N/A | NHD ^6^ |  | **0** | **1** | **-11.90** | **-0.83** | **-3.41** | **0.95** | **26** | **0.98** |
| C51Y | N/A | N/A | N/A |  | **0** | **1** | **-9.68** | **-0.93** | 0.16 | **0.94** | N/A | N/A |
| R52H | N/A | N/A | AD ^7^ |  | **0** | **1** | **-4.14** | **-0.67** | -0.51 | **0.72** | N/A | N/A |
| R62H | LB | N/A | AD ^2^ | ^3^ | 0.37 | 0.04 | -0.13 | **-1.75** | -0.11 | 0.13 | 14.96 | 0.022 |
| T66M | LB | LB | NHD ^2^ | ^3,^ ^4^ | **0** | **0.98** | **-3.57** | **-0.52** | -0.22 | **0.43** | **26.1** | **0.88** |
| T85K | N/A | N/A | N/A |  | 0.04 | 0.32 | -0.62 | -0.4 | 1.91 | 0.32 | 9.51 | 0.45 |
| D87N | LB | N/A | AD ^2^ | ^3^ | 0.43 | **1** | -2.06 | **-1.84** | 1.63 | 0.43 | **20.5** | **0.51** |
| T96K | IB | N/A | AD ^2^ | ^2^ | **0** | **0.99** | **-4.49** | **-2.16** | -0.18 | **0.80** | **24.2** | **0.91** |
| R98W | N/A | N/A | AD ^8^ |  | **0** | 0.60 | **-4.25** | **-0.95** | -0.24 | 0.30 | **22.5** | **0.65** |
| R98Q | N/A | N/A | N/A |  | 0.16 | 0.00 | -0.87 | **-1.48** | N/A | 0.21 | N/A | N/A |
| A105V | N/A | N/A | N/A |  | **0** | **0.97** | **-3.34** | 0.03 | -0.21 | 0.31 | **21.8** | **0.86** |
| V126G | N/A | LB | NHD ^2^ | ^4^ | **0** | **0.99** | **-5.25** | **-1.76** | 0.81 | **0.91** | **27.7** | **0.91** |

Here, N/A: Not available, MDS: Molecular Dynamics Simulation, LB: Loss of Binding, IB: Increase of Binding, FTD: Frontotemporal Dementia, NHD: Nasu-Hakola disease, AD: Alzheimer's Disease. In *in silico* analysis, the scores in Bold form indicate deleterious or damaging, according to the individual criteria, i.e. SIFT (= 0), PolyPhen‐2 (>0.9), PROVEAN (<−2.5), I‐Mutant 3.0 (<−0.5), FATHMM (<-3.0 or >3.0), MutPred (>0.75), CADD (>20) and Condel (>0.8).

**Table S2.** The summery of MD trajectory analysis in terms of RMSD, Rg, SASA, and secondary structural elements throughout the protein structure in different simulation systems.

| **Name of System** | **RMSD**  **(Å)** | **Rg**  **(Å)** | **SASA**  **(nm^2^)** | **% of Helix** | **% of Strand** | **% of Total SSE** |
| --- | --- | --- | --- | --- | --- | --- |
| Wild | 1.28±0.001 | 13.68 ±0.001 | 65.62±0.117 | 0.40 | 46.97 | 47.37 |
| Y38C | 1.29±0.001 | 13.66±0.001 | 65.31±0.121 | 1.47 | 46.97 | 48.43 |
| W50C | 1.67±0.001 | 13.73±0.001 | 65.77±1.168 | 0.16 | 47.44 | 47.60 |
| T66M | 1.781±0.001 | 13.68±0.001 | 65.71±0.122 | 0.93 | 48.80 | 48.80 |
| V126G | 1.930±0.004 | 13.65±0.001 | 66.34±0.118 | 1.35 | 47.90 | 49.25 |

**
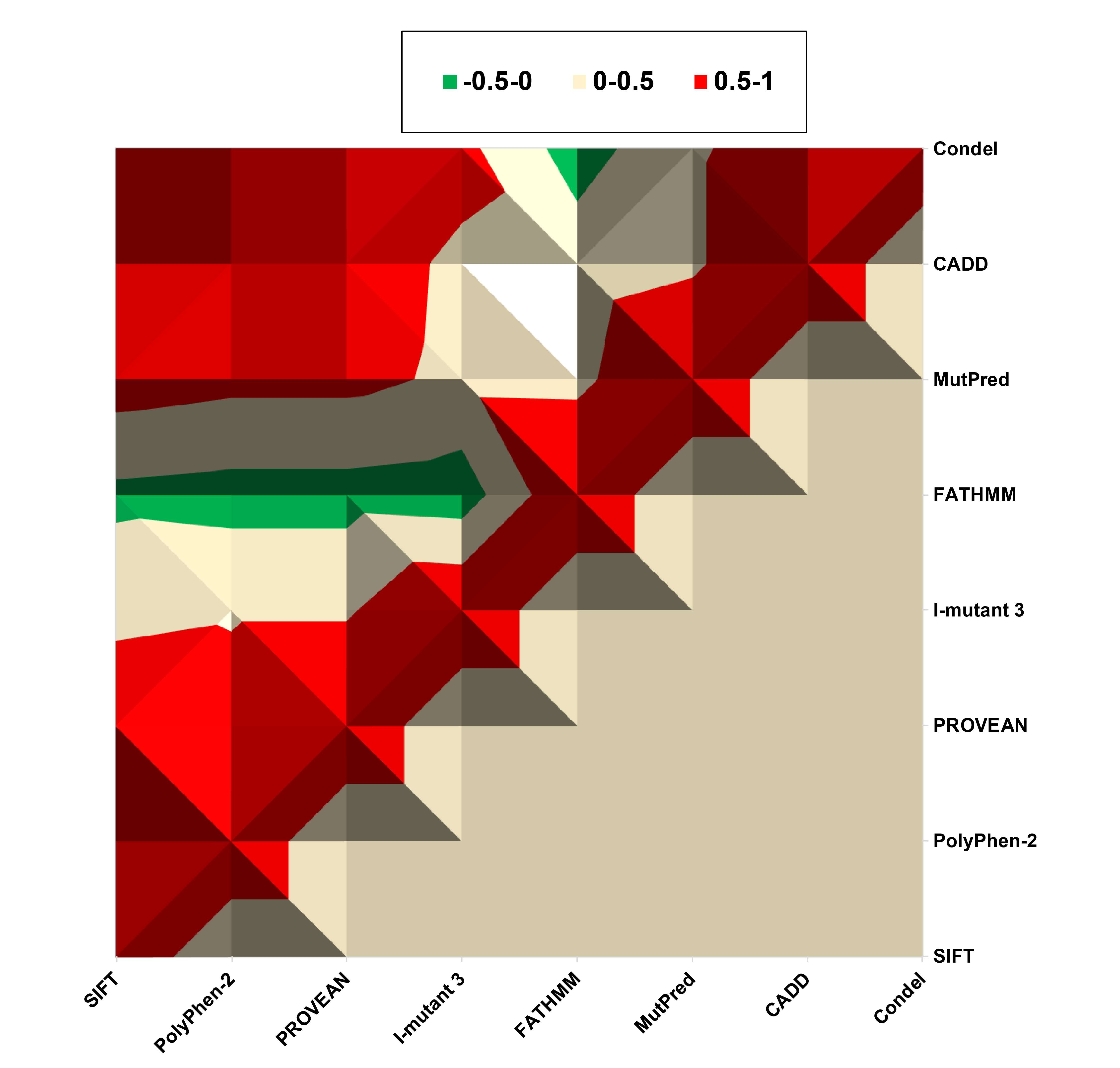
**

**Figure S1:** The correlations among the deleterious predictions by various computational tools in TREM2 gene is represented in a surface chart. Here darker red region indicates positive correlation, while green color describes anti-correlation.


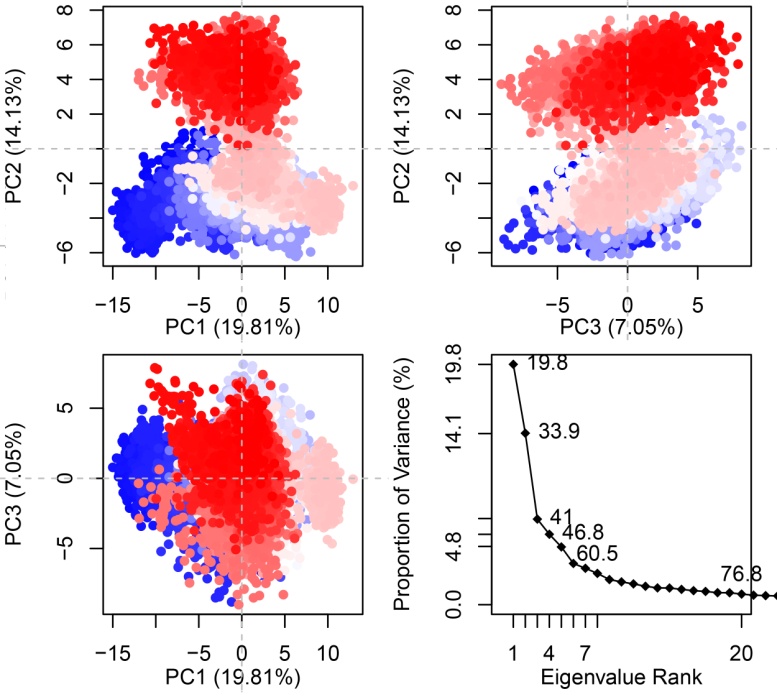

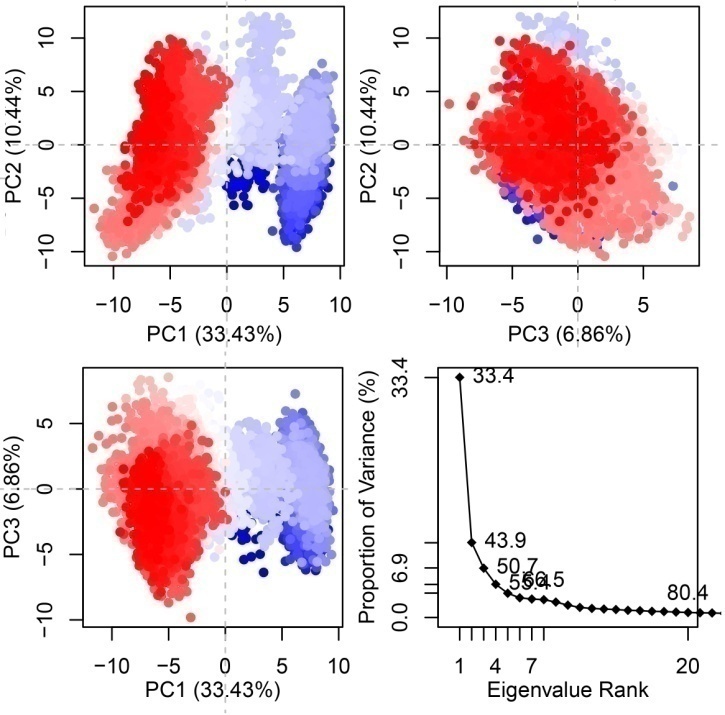

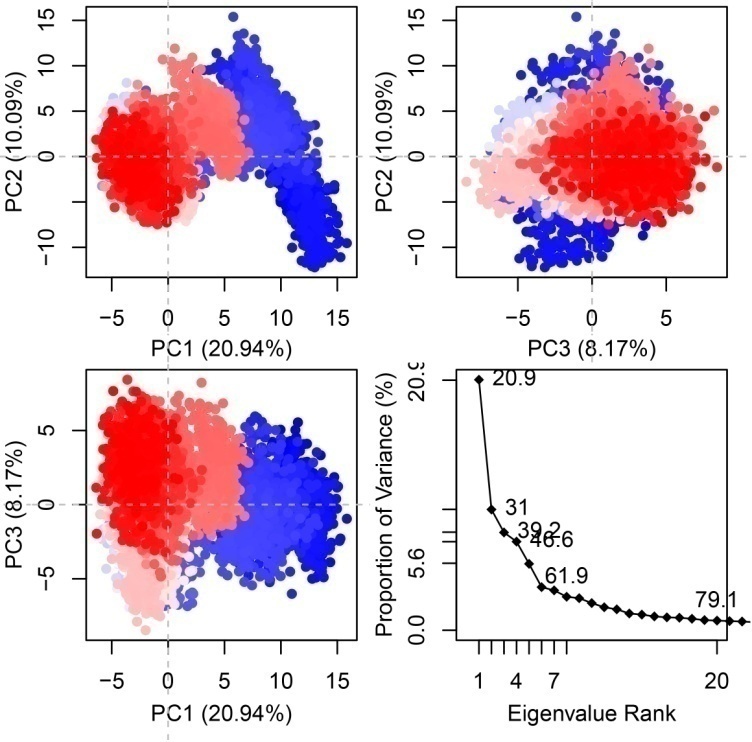

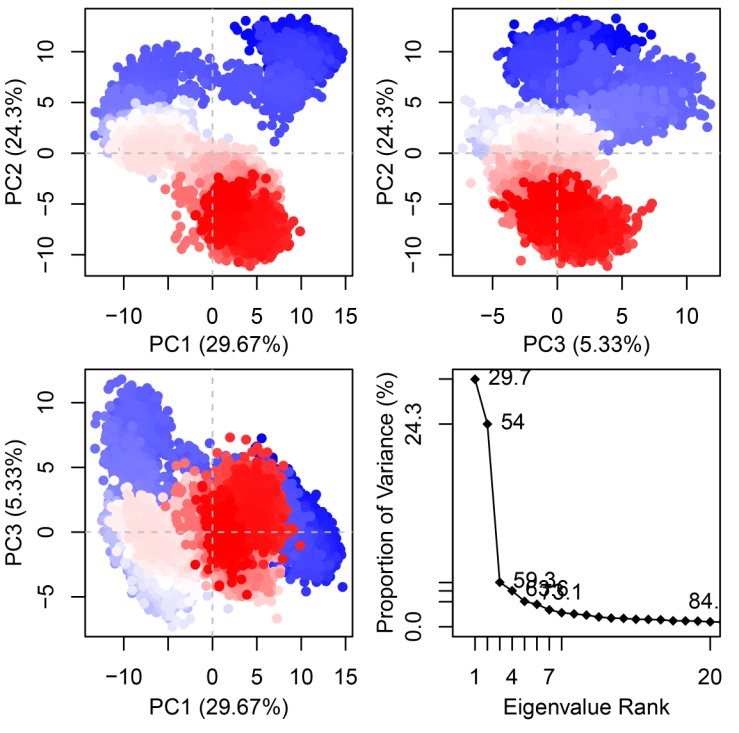


**a)**

**b)**

**c)**

**d)**


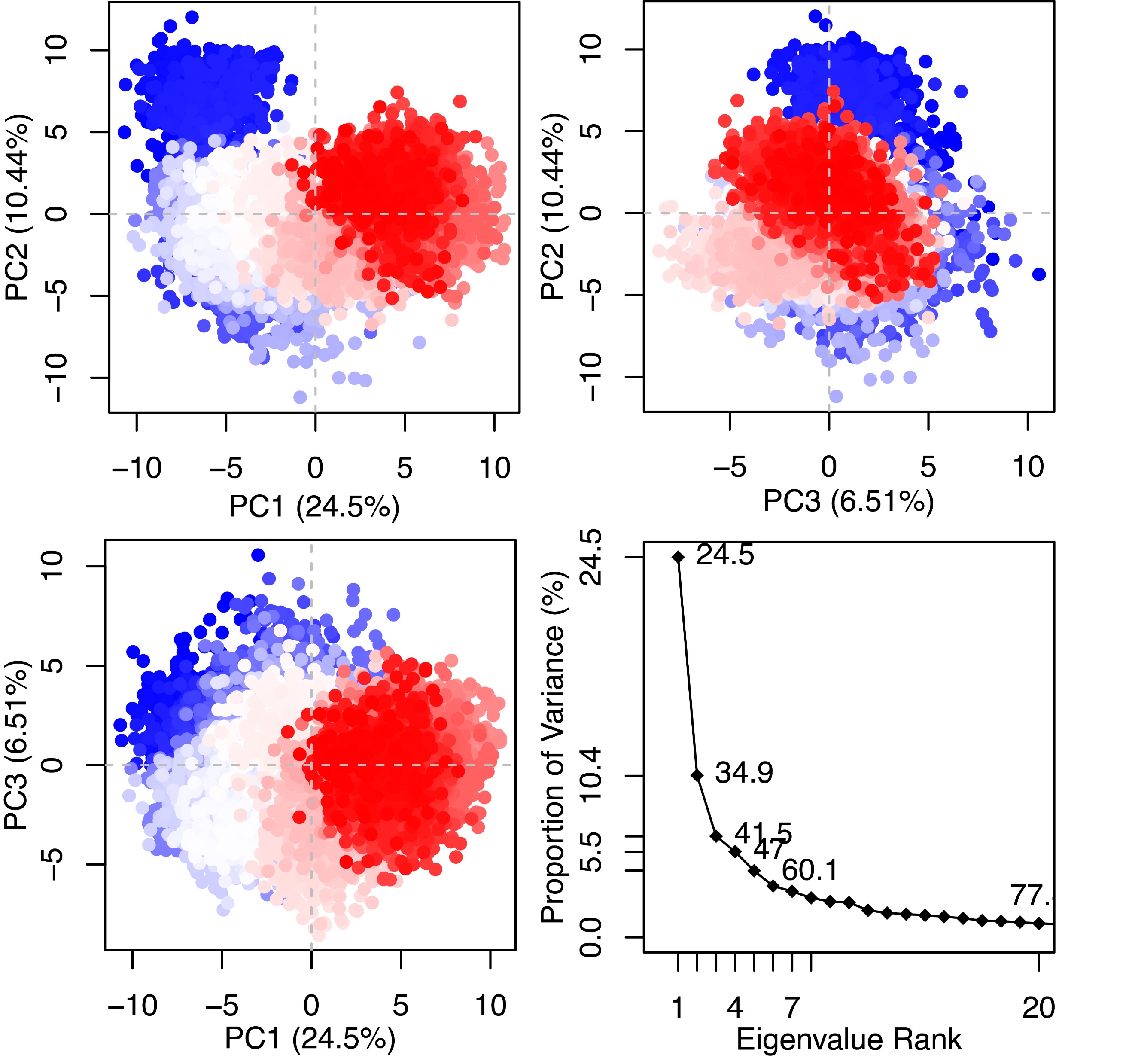


**e)**

**Figure S2.** Principle Component Analysis (PCA) regarding the TREM2 protein in four different systems, wild (a), Y38C (b), W50C (c), T66M (d), and V126G (e) structure.


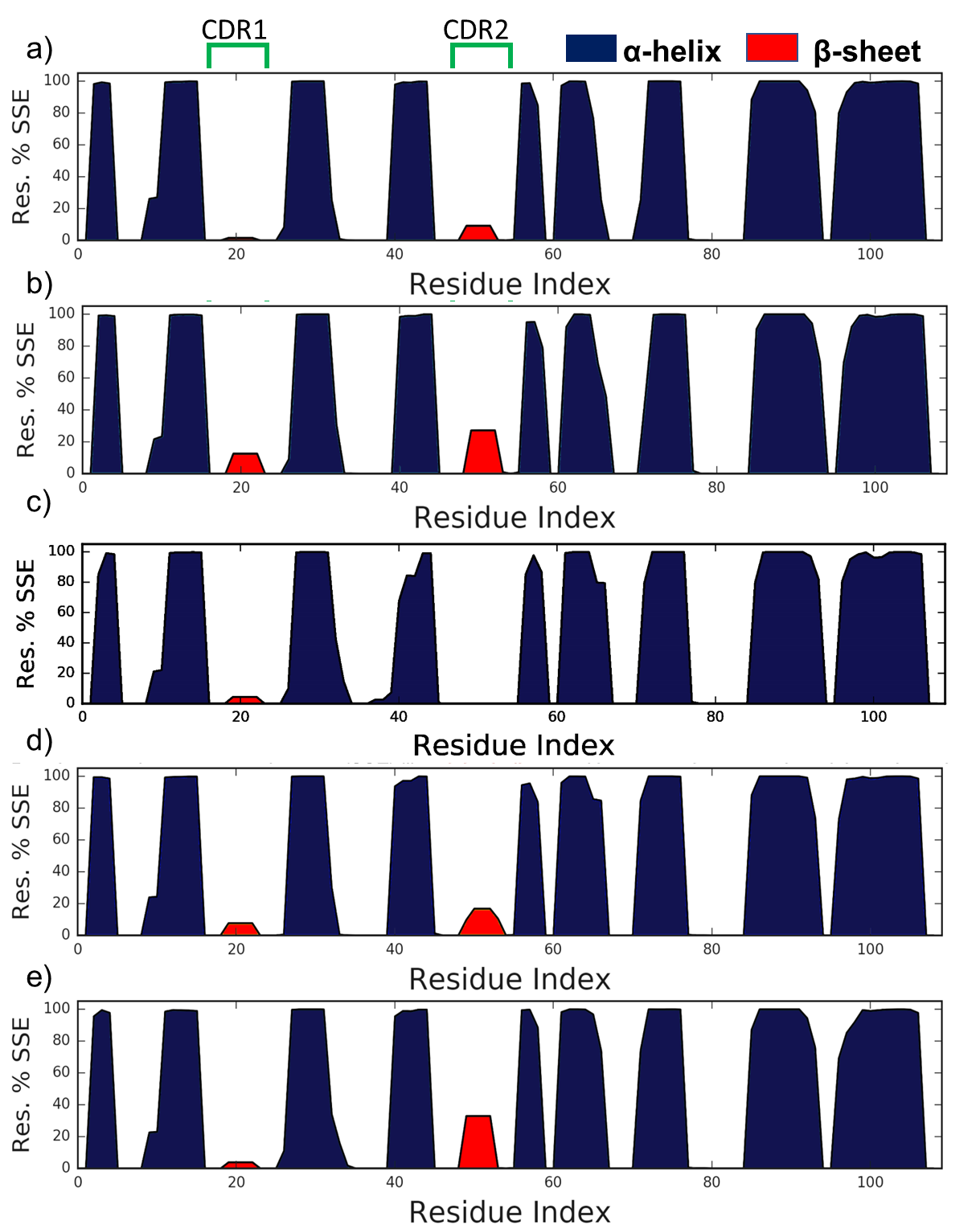


**Figure S3.** Residue-specific average secondary structural preferences for wild (a), Y38C (b), W50C (c), T66M (d), and V126G (e) structure, during 100 ns simulation. Here, dark blue color stands for Beta sheets and red color for alpha helix conformation.


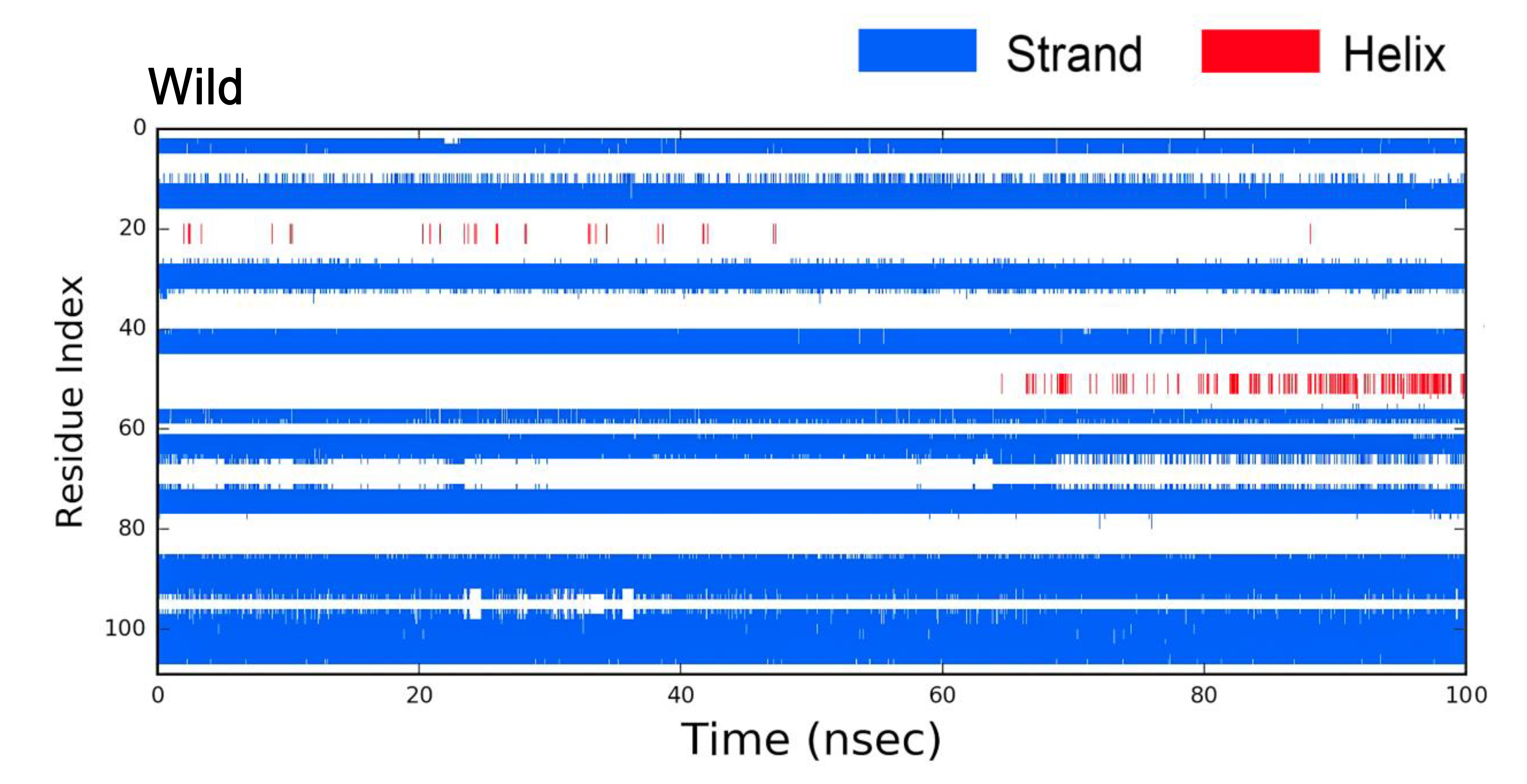


**Figure S4.** Residue-specific average secondary structural elements of wild structure along with the total simulation time. The occupancy in white color represents loop, helices in red, while strands in blue.


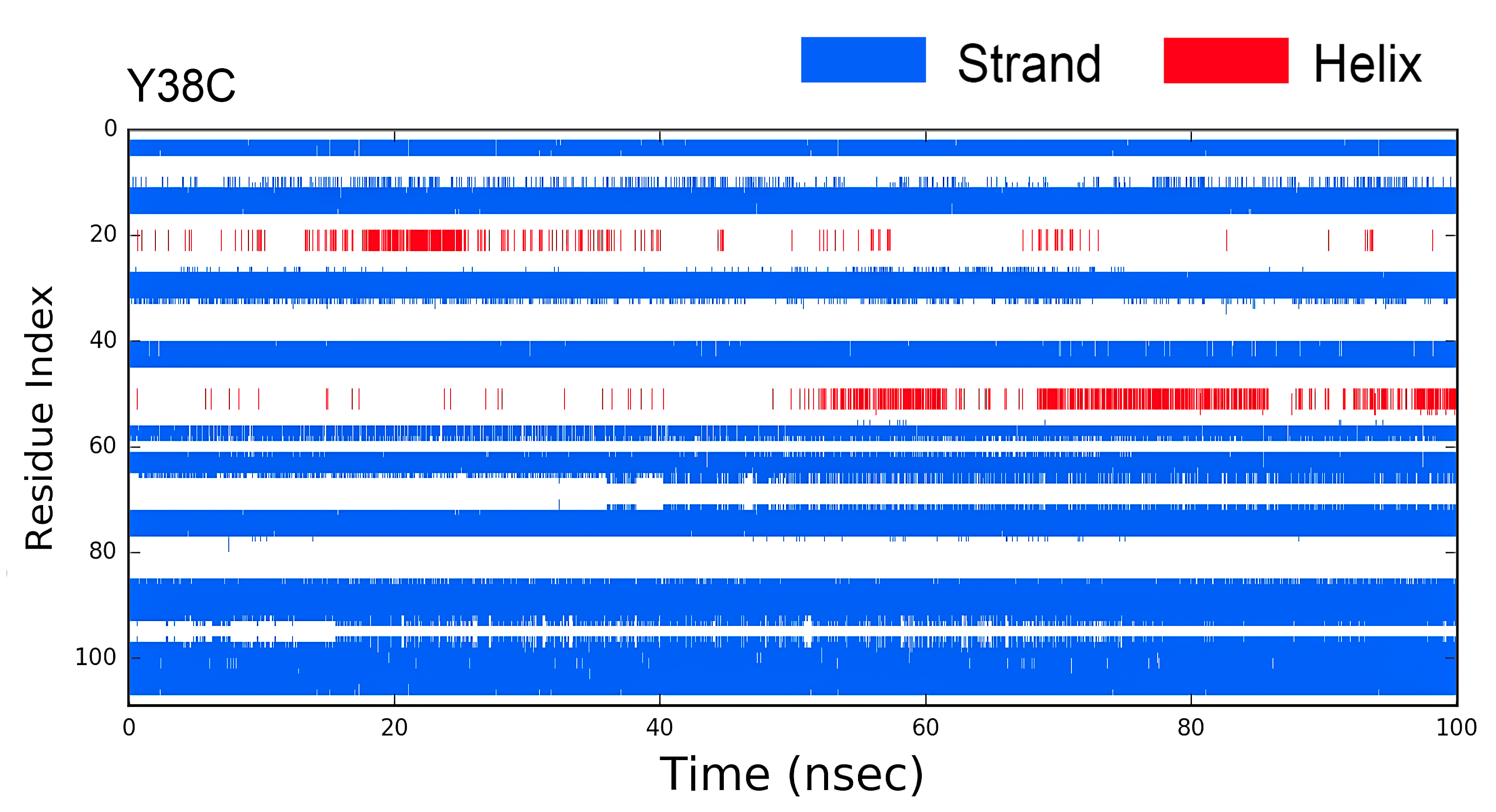


**Figure S5.** Residue-specific average secondary structural elements of Y38C mutant structure along with the total simulation time. The occupancy in white color represents loop, helices in red, while strands in blue.


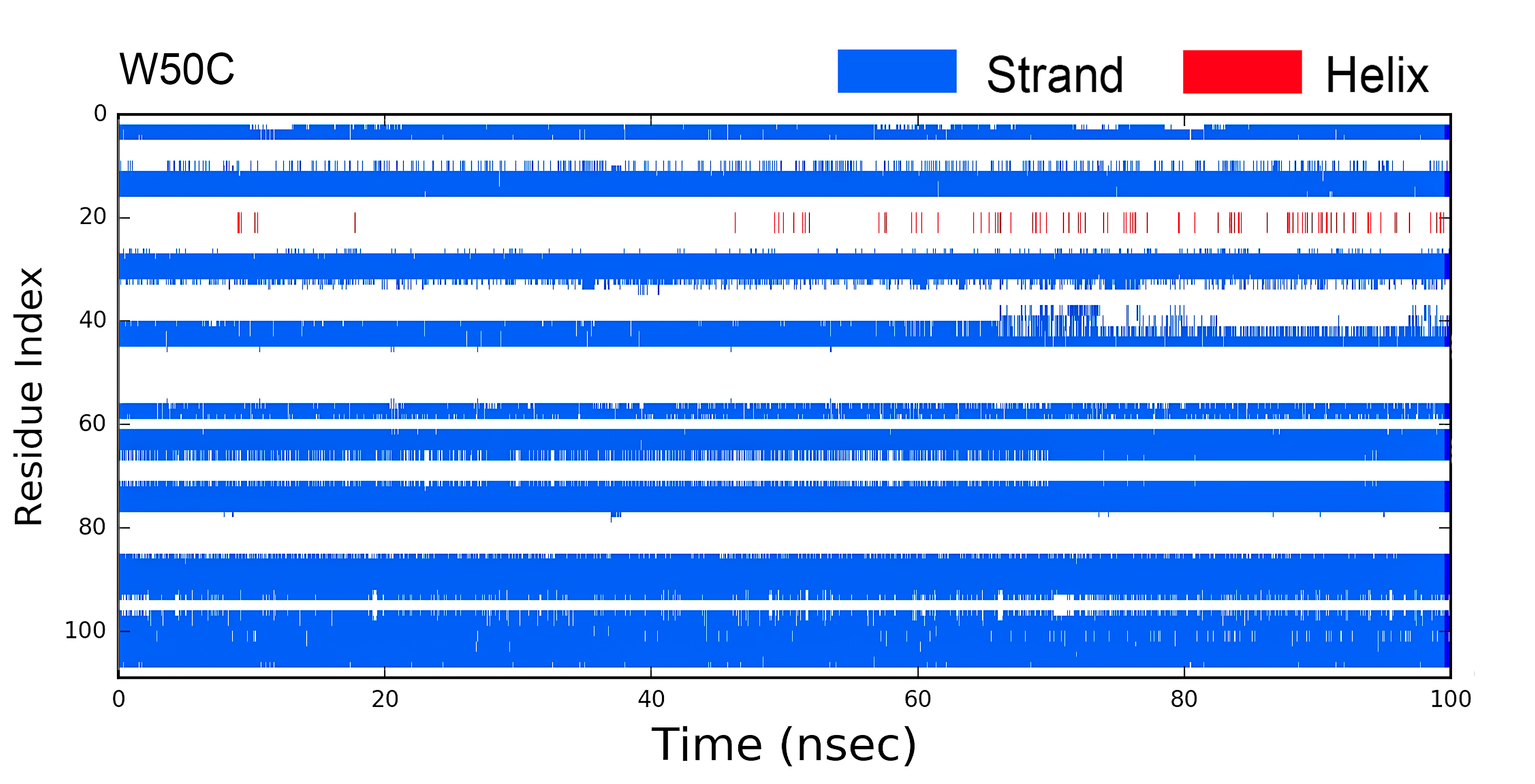


**Figure S6.** Residue-specific average secondary structural elements of W50C mutant structure along with the total simulation time. The occupancy in white color represents loop, helices in red, while strands in blue.


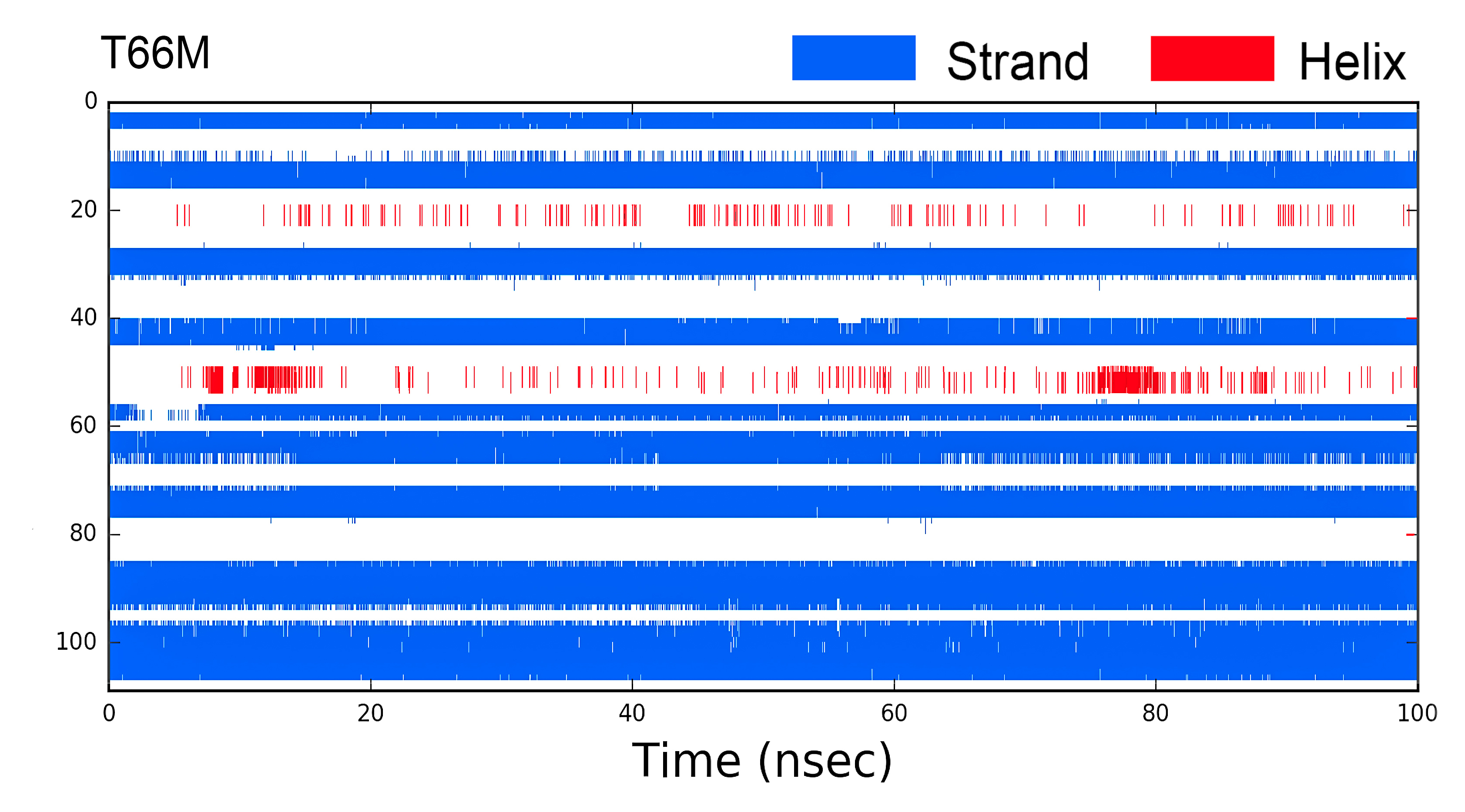


**Figure S7.** Residue-specific average secondary structural elements of T66M mutant structure along with the total simulation time. The occupancy in white color represents loop, helices in red, while strands in blue.


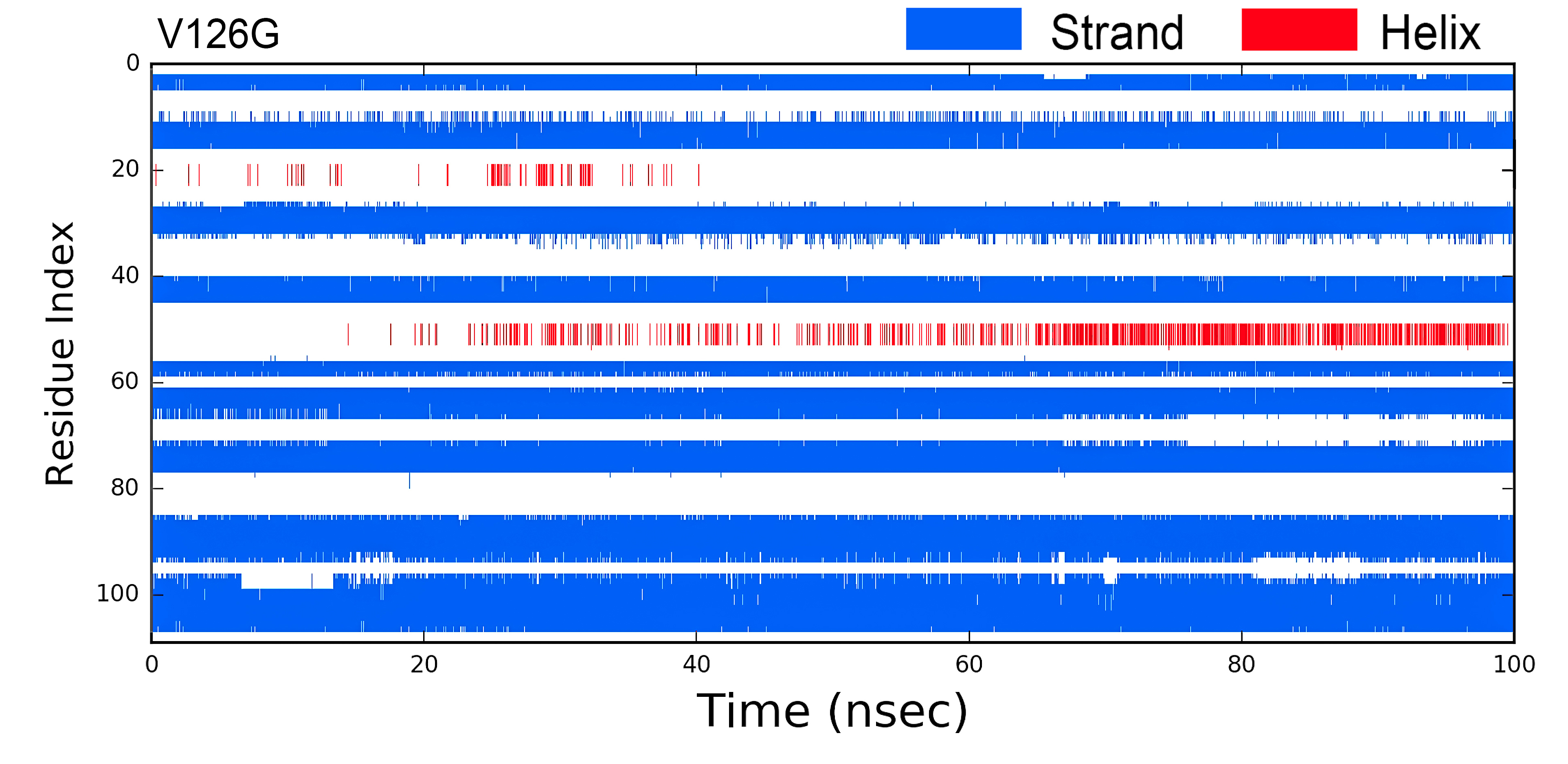


**Figure S8.** Residue-specific average secondary structural elements of V126G mutant structure along with the total simulation time. The occupancy in white color represents loop, helices in red, while strands in blue.

**Figure S9.** Hydrogen bond occupancy analysis for within the CDR1 (a), CDR2 (b), between the CDR1 and CDR2 loop (c).

**Figure S10.** Representation of residue interaction network (RIN) for wild (a), Y38C (b), W50C (c), T66M (d), and V126G (e) system, considering the 1000 representative structures from the 100 ns of MD, with trajectory interval of 100 ps. Here the red node indicates helices, blue indicates β-sheets, and gray indicates the loop secondary structures. In addition, the edge with blue line represents the contact between the main chain (dark blue) and side chain (light blue) interactions, whereas red color represents hydrogen bonding with main chain (dark red) and side chain (light red), respectively.

**References**

1 Thelen, M. *et al.* Investigation of the role of rare TREM2 variants in frontotemporal dementia subtypes. *Neurobiology of aging* **35**, 2657. e2613-2657. e2619 (2014).

2 Kober, D. L. *et al.* Neurodegenerative disease mutations in TREM2 reveal a functional surface and distinct loss-of-function mechanisms. *Elife* **5**, e20391 (2016).

3 Yeh, F. L., Wang, Y., Tom, I., Gonzalez, L. C. & Sheng, M. TREM2 binds to apolipoproteins, including APOE and CLU/APOJ, and thereby facilitates uptake of amyloid-beta by microglia. *Neuron* **91**, 328-340 (2016).

4 Dash, R., Choi, H. J. & Moon, I. S. Mechanistic insights into the deleterious role of nasu-hakola disease associated TREM2 variants. *bioRxiv*, 705608 (2019).

5 Cuyvers, E. *et al.* Investigating the role of rare heterozygous TREM2 variants in Alzheimer's disease and frontotemporal dementia. *Neurobiology of aging* **35**, 726. e711-726. e719 (2014).

6 Dardiotis, E. *et al.* A novel mutation in TREM2 gene causing Nasu-Hakola disease and review of the literature. *Neurobiology of aging* **53**, 194. e113-194. e122 (2017).

7 Jin, S. C. *et al.* Coding variants in TREM2 increase risk for Alzheimer's disease. *Human molecular genetics* **23**, 5838-5846 (2014).

8 Guerreiro, R. *et al.* TREM2 variants in Alzheimer's disease. *New England Journal of Medicine* **368**, 117-127 (2013).
